# Fixed-Dose Rifapentine–Isoniazid (1HP) for Tuberculosis Preventive Treatment in Chinese Adults: A Prospective Real-World Safety and Pharmacokinetic Study

**DOI:** 10.1093/cid/ciag247

**Published:** 2026-04-13

**Authors:** Senlin Zhan, Weijian Liu, Liangzi Yang, Zijiao Chen, Tian He, Guofang Deng, Hongjuan Qin, Wei Li, Peize Zhang

**Affiliations:** Department of Pulmonary Medicine & Tuberculosis, The Third People's Hospital of Shenzhen, National Clinical Research Center for Infectious Disease, Southern University of Science and Technology, Shenzhen, Guangdong Province, China; Shenzhen Clinical Research Center for Tuberculosis, Shenzhen, Guangdong Province, China; Department of Pulmonary Medicine & Tuberculosis, The Third People's Hospital of Shenzhen, National Clinical Research Center for Infectious Disease, Southern University of Science and Technology, Shenzhen, Guangdong Province, China; Shenzhen Clinical Research Center for Tuberculosis, Shenzhen, Guangdong Province, China; Department of Pulmonary Medicine & Tuberculosis, The Third People's Hospital of Shenzhen, National Clinical Research Center for Infectious Disease, Southern University of Science and Technology, Shenzhen, Guangdong Province, China; Shenzhen Clinical Research Center for Tuberculosis, Shenzhen, Guangdong Province, China; Department of Pulmonary Medicine & Tuberculosis, The Third People's Hospital of Shenzhen, National Clinical Research Center for Infectious Disease, Southern University of Science and Technology, Shenzhen, Guangdong Province, China; Shenzhen Clinical Research Center for Tuberculosis, Shenzhen, Guangdong Province, China; Department of Pulmonary Medicine & Tuberculosis, The Third People's Hospital of Shenzhen, National Clinical Research Center for Infectious Disease, Southern University of Science and Technology, Shenzhen, Guangdong Province, China; Department of Pharmacy, The Third People's Hospital of Shenzhen, National Clinical Research Center for Infectious Disease, Southern University of Science and Technology, Shenzhen, Guangdong Province, China; Department of Pulmonary Medicine & Tuberculosis, The Third People's Hospital of Shenzhen, National Clinical Research Center for Infectious Disease, Southern University of Science and Technology, Shenzhen, Guangdong Province, China; Shenzhen Clinical Research Center for Tuberculosis, Shenzhen, Guangdong Province, China; Department of Pulmonary Medicine & Tuberculosis, The Third People's Hospital of Shenzhen, National Clinical Research Center for Infectious Disease, Southern University of Science and Technology, Shenzhen, Guangdong Province, China; Shenzhen Clinical Research Center for Tuberculosis, Shenzhen, Guangdong Province, China; Department of Pulmonary Medicine & Tuberculosis, The Third People's Hospital of Shenzhen, National Clinical Research Center for Infectious Disease, Southern University of Science and Technology, Shenzhen, Guangdong Province, China; Department of Pharmacy, The Third People's Hospital of Shenzhen, National Clinical Research Center for Infectious Disease, Southern University of Science and Technology, Shenzhen, Guangdong Province, China; Department of Pulmonary Medicine & Tuberculosis, The Third People's Hospital of Shenzhen, National Clinical Research Center for Infectious Disease, Southern University of Science and Technology, Shenzhen, Guangdong Province, China; Shenzhen Clinical Research Center for Tuberculosis, Shenzhen, Guangdong Province, China

**Keywords:** tuberculosis infection, tuberculosis preventive treatment, rifapentine, pharmacokinetics, fixed-dose regimen

## Abstract

**Background:**

The World Health Organization recommends 1 month of daily rifapentine plus isoniazid (1HP) for tuberculosis preventive treatment (TPT). Real-world safety and feasibility data remain scarce, particularly for East Asian populations, and the performance of fixed-dose 1HP in routine clinical practice requires prospective evaluation.

**Methods:**

We conducted a prospective study at a tuberculosis referral center in Shenzhen, China, enrolling 136 non-human immunodeficiency virus adults (body weight 42–90 kg) with tuberculosis infection between October 2024 and July 2025. All participants received fixed-dose 1HP. Outcomes included treatment completion (defined as taking ≥23 doses within 40 days), adverse events graded by Common Terminology Criteria for Adverse Events (CTCAE) v5.0, and 12-hour postdose rifapentine concentrations measured by liquid chromatography-tanden mass spectrometry.

**Results:**

Treatment completion reached 76.5% (104/136; 95% confidence interval [CI], 68.7%–82.8%). Adverse events occurred in 26.5% of participants, most commonly neutropenia (14.7%) and rash (6.6%); grade ≥ 3 events were observed in only 1.5% (2/136), both in patients receiving concurrent methotrexate. Median rifapentine concentration was 21.8 mg/L (interquartile range [IQR], 14.8–28.4), and 91.9% of participants exceeded the model-informed 10 mg/L threshold. Body weight demonstrated a modest inverse correlation with rifapentine concentrations (ρ = −0.24; *P* = .005), accounting for only 7.1% of interindividual variability.

**Conclusions:**

Fixed-dose 1HP exhibited good tolerability and achieved acceptable completion rate in Chinese adult population. Pharmacokinetic results confirmed adequate rifapentine exposure across the studied weight range, lending support to fixed-dose 1HP for programmatic scale-up.

## Teaser

Among 136 Chinese adults enrolled in a prospective implementation study, fixed-dose 1HP achieved 76.5% completion with few severe events (1.5% grade ≥ 3). Rifapentine exposure was adequate in 91.9% of participants across the 42–90 kg weight range, supporting simplified fixed dose.

Tuberculosis (TB) is the leading infectious killer worldwide, with an estimated 10.7 million people developing TB in 2024 [1]. China accounts for 6.5% of the global TB burden, with approximately 695 000 people diagnosed with TB [1]. Despite this substantial disease burden, coverage of tuberculosis preventive treatment (TPT) remains limited: Fewer than 20% of eligible household contacts initiate TPT, and coverage varies across regions [2]. Short-course regimens may help close this implementation gap. Beyond household contacts, indications for TPT have expanded to include transplant candidates, patients initiating immunosuppressive therapy, and women preparing for in vitro fertilization–embryo transfer (IVF-ET) in high-incidence settings. Implementation data for these diverse clinical populations remain sparse.

Tuberculosis preventive treatment represents a cornerstone of the WHO End TB Strategy. Conventional 6–9-month isoniazid monotherapy achieves only 50%–70% completion in programmatic settings, limited by cumulative hepatotoxicity, pill burden, and the extended treatment duration [3, 4]. Rifapentine-based short-course regimens offer more practical alternatives. Both 3 months of weekly rifapentine–isoniazid (3HP) and 1 month of daily rifapentine–isoniazid (1HP) demonstrated noninferiority to 9-month isoniazid in randomized trials [5, 6]. Completion rates reported from these direct observed therapy (DOT) trials have been remarkably high—97% in BRIEF-TB among people with human immunodeficiency virus (HIV) (PWH) [6] and 82.9% in a Taiwanese non-HIV cohort [7]. The 2024 World Health Organization (WHO) guidelines include 1HP as one of the recommended options for TPT [8]. However, real-world evidence on the safety and completion rate of 1HP, which typically relies on self-administered therapy (SAT) in diverse populations, is lacking.

Current guidelines recommend daily weight-based rifapentine doses—300 mg (<35 kg), 450 mg (35–45 kg), and 600 mg (>45 kg)—but population pharmacokinetic modeling by Hibma et al suggests that a fixed 600 mg dose may achieve adequate exposure across a wide range of body weights [9]. If validated prospectively, fixed-dose 1HP could simplify administration and streamline supply chains. East Asian populations might be particularly relevant for such validation, given their lower median body weight [6]. Moreover, arylacetamide deacetylase (AADAC) polymorphisms that influence rifapentine clearance exhibit different allele frequencies in East Asian compared with Western populations [10]. Recent prospective data from Chinese children indicated that weight-based dose 1HP might lead to inadequate rifapentine exposure [11, 12]. These factors underscore the need for prospective validation of fixed-dose 1HP in East Asian adults.

To address these gaps, we conducted a prospective study at a large tuberculosis center in Shenzhen, China, enrolling adults with a broad range of TPT indications encountered in routine practice. The study aimed to (1) evaluate the safety and tolerability of fixed-dose 1HP in a real-world setting, (2) establish completion rate benchmarks for programmatic planning, and (3) confirm that a fixed 600 mg rifapentine dose provides adequate drug exposure across a body weight range of 42–90 kg. By including diverse participants, we sought to generate evidence applicable to real-world TPT implementation.

## METHODS

### Study Design and Participants

We conducted an open-label, prospective, single-arm study to evaluate the safety, tolerability, completion rate, and rifapentine pharmacokinetics of the 1HP regimen in adults with tuberculosis infection (TBI) at high risk of progression to active tuberculosis. The study was performed at a 1500-bed tertiary referral hospital in Shenzhen, China, which serves as the designated municipal tuberculosis treatment facility for a population of 17.5 million. Enrollment began on 1 October 2024 and is expected to continue until 1 October 2026. The study protocol was approved by the Institutional Ethics Committee (Approval No. 2024-196-02) and registered at the Chinese Clinical Trial Registry (ChiCTR2400089896). All participants provided written informed consent. The study adhered to Strengthening the Reporting of Observational Studies in Epidemiology (STROBE) guidelines for observational cohort studies [13].

### Participants and Eligibility

Eligible participants were adults (≥18 years) with confirmed TBI (positive interferon-gamma release assay [IGRA]) and no evidence of active tuberculosis (based on symptom screening, chest radiography, and, when indicated, computed tomography). Detailed inclusion and exclusion criteria are provided in Supplementary Table 1.

### Sample Size and Recruitment

The study initially aimed to enroll 60 non-HIV participants and 20 PWH to evaluate safety and rifapentine concentrations. After the first 60 non-HIV participants had completed treatment and an internal safety review confirmed no unexpected adverse events, the sample size for non-HIV participants was expanded to 130 to allow a more precise estimate of the treatment completion rate (using an expected completion rate of 80%, with an expected 95% confidence interval (CI) width of approximately ±8.5% points, calculated as 1.96 × √[*P*{1−*P*}/n]). As of 30 July 2025, 136 non-HIV participants had been enrolled and included in the analysis. Enrollment of PWH is still ongoing. All participants were recruited consecutively from the hospital's tuberculosis clinic. No changes were made to eligibility criteria during the expansion; the expanded cohort represents the same underlying population.

### Study Procedures and Follow-Up

Participants received fixed-dose rifapentine 600 mg plus isoniazid 300 mg once daily for 28 days without weight-based adjustment. Medications were self-administered, along with standardized counseling to take doses with food to enhance rifapentine absorption [14]. Weekly follow-up visits included adherence assessment through pill counts and self-report, systematic symptom review using a checklist covering constitutional, gastrointestinal, dermatologic, and hematologic domains and documentation of concomitant medications. Laboratory evaluations comprised complete blood count, liver and kidney function, and rifapentine pharmacokinetics.

### Pharmacokinetic Sampling

Steady state was defined as at least 5 elimination half-lives; for rifapentine (half-life 13–19 hours), this corresponds to day 7. Venous blood samples (3–5 mL in Ethylenediaminetetraacetic acid tubes) were collected at weekly visits during weeks 1–4, targeting 12 hours postdose (acceptable window 10–14 hours) to approximate trough concentrations. This midinterval sampling was selected as a surrogate for overall exposure because: (1) rifapentine exhibits linear elimination, allowing a single concentration during the postdistribution phase to correlate with overall drug exposure; and (2) intensive serial sampling was not feasible in an outpatient setting. Samples were centrifuged immediately (3000 rpm, 10 minutes), and plasma was stored at −80°C until analysis.

Rifapentine and 25-desacetyl-rifapentine plasma concentrations were quantified using a validated liquid chromatography-tandem mass spectrometry assay (Shimadzu LC-MS 8050, Kyoto, Japan). Methodological details and assay validation parameters appear in Supplementary Methods 1 and in our companion pediatric study [11]. Rifapentine-d3 served as the internal standard. The lower limit of quantification was 0.1 mg/L, with interassay coefficients of variation below 10% across the 0.1–100 mg/L calibration range.

### Outcomes Definition

According to the guidance of WHO [8], treatment completion was defined as taking ≥23 prescribed doses within 40 days in our study. Adverse events were graded according to Common Terminology Criteria for Adverse Events (CTCAE) version 5.0 [15]. Neutropenia was classified as grade 1 (below the lower limit of normal to 1.5 × 10^9^/L), grade 2 (below 1.5–1.0 × 10^9^/L), grade 3 (below 1.0–0.5 × 10^9^/L), and grade 4 (below 0.5 × 10^9^/L). Population rifapentine pharmacokinetic data simulated by Hibma et al demonstrated that a rifapentine AUC_0_–_24_ of approximately 350 mg·h/L was associated with optimal bactericidal activity. Based on rifapentine's linear pharmacokinetics and long elimination half-life, a concentration of 10 mg/L corresponds to this target area under curve (AUC) [9]. Thus, a concentration threshold of 10 mg/L or greater was used as a model-informed exploratory benchmark in our study.

### Statistical Analysis

Categorical variables were summarized as frequencies and percentages, and continuous variables as medians with interquartile ranges. Completion rates with 95% CIs were calculated using the Wilson score method.

For the primary analysis examining the association between body weight and rifapentine concentration, we used each participant's median trough concentration ( ampa#thinsp;= 136) to ensure independence, as individuals contributed multiple samples. Spearman's rank correlation coefficient (ρ) assessed nonparametric associations, and variance explained was quantified using the coefficient of determination (R^2^) from ordinary least squares regression. Fisher's exact test was applied for categorical comparisons given small subgroup sizes.

Given the study's implementation focus, consistency across participants with different TPT indications was expected; meaningful heterogeneity would have suggested limited generalizability. Statistical procedures are detailed in Supplementary Methods 2.

Given the descriptive study design, no adjustments were made for multiple comparisons. *P* values are nominal and represent measures of association strength rather than definitive hypothesis testing. Analyses were performed using R version 4.3.1 (R Foundation for Statistical Computing, Vienna, Austria).

## RESULTS

### Participant Characteristics

**Figure 1.**
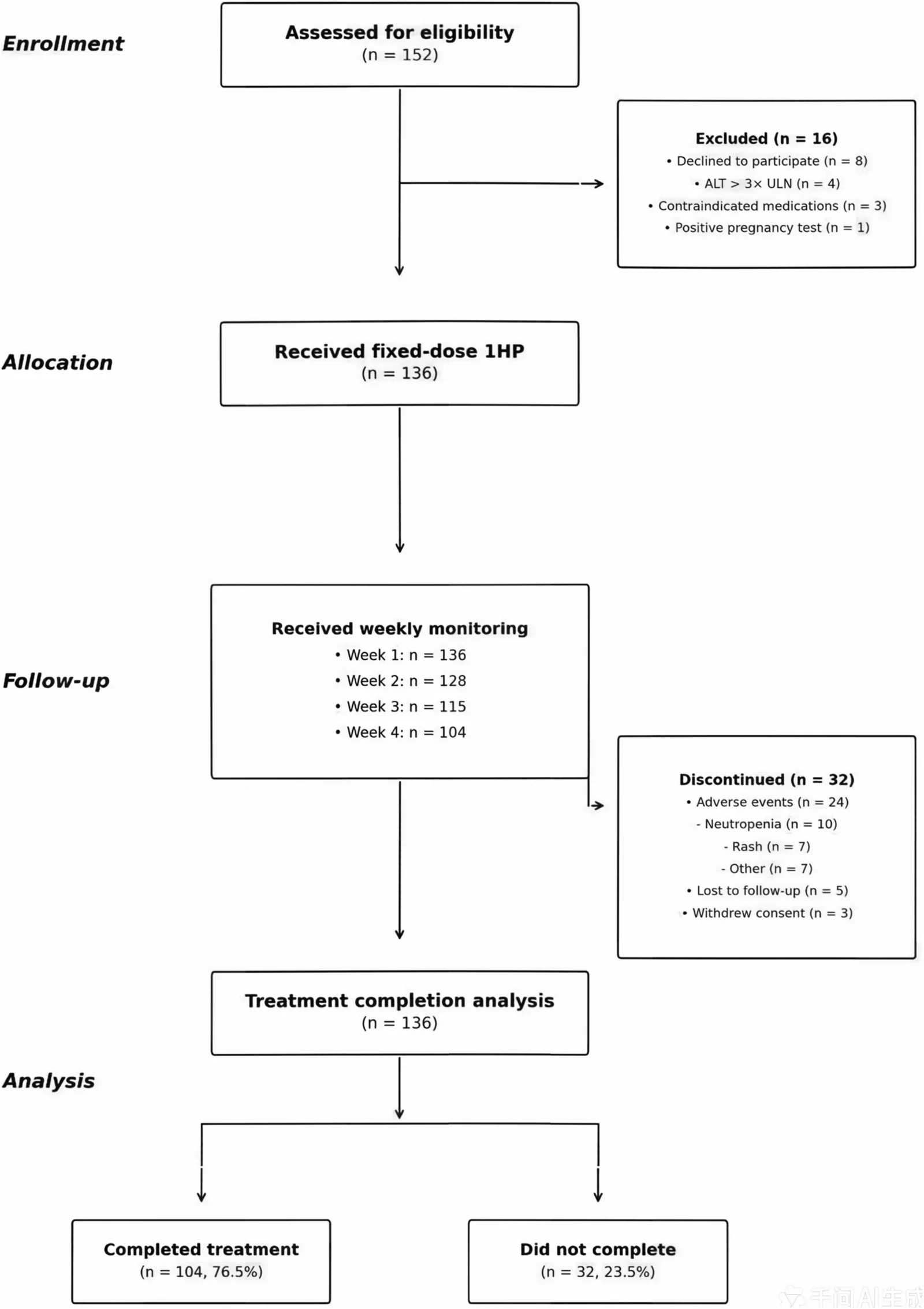
Participant flow diagram. Of 152 individuals assessed for eligibility during the enrollment period (June 2022–December 2023), 16 were excluded: 8 declined participation, 4 had baseline alanine aminotransferase greater than 3 times the upper limit of normal, 3 were receiving contraindicated medications, and 1 had a positive pregnancy test. The remaining 136 participants received fixed-dose 1HP and constituted the safety analysis population. Treatment completion was 76.5% (104/136).

From 1 October 2024 to 30 July 2025, 152 individuals were assessed for eligibility. Sixteen (10.5%) were excluded: 8 declined participation, 4 had baseline alanine aminotransferase exceeding 3 times the upper limit of normal, 3 were receiving contraindicated medications, and 1 had a positive pregnancy test. The remaining 136 participants initiated 1HP and comprised the safety analysis population (Figure 1).

Baseline characteristics are presented in Table 1. The median age was 38 years (interquartile range [IQR], 34–49), with 64.0% (87/136) female. The median body weight was 60.0 kg (IQR, 55.0–69.0; range, 42.0–90.0), and the median body mass index was 23.0 kg/m^2^ (IQR, 21.5–24.7). Tuberculosis preventive treatment indications included household contact (39.7%), immunosuppressive therapy recipients and candidate (58.1%), and overlapping indications (2.2%). Six participants had baseline immunocompromise: 1 was under observation for lymphoma, and the remaining 5 had rheumatic arthritis. Among them, 3 participants were taking methotrexate-based regimens (2 without concurrent folic acid supplementation and 1 receiving weekly folic acid 5 mg); the other 2 participants were receiving prednisone. Of the participants, 23.5% (32/136) were women with a history of 2 or more failed IVF-ET cycles who were planned to undergo immunotherapy.

**Table 1.** Baseline Characteristics of Study Participants (N = 136)

Characteristic	Value
Age, years, median (IQR)	38 (34–49)
Female sex, n (%)	87 (64.0)
Body weight, kg, median (IQR)	60.0 (55.0–69.0)
Body mass index, kg/m^2^, median (IQR)	23.0 (21.5–24.7)
Weight category, n (%)	
<55 kg	29 (21.3)
55–65 kg	56 (41.2)
>65 kg	51 (37.5)
TPT indication, n (%)	
Household contact	54 (39.7)
Immunosuppressive therapy recipients and candidate	79 (58.1)
Both indications	3 (2.2)
IVF-ET status, n (%)	
IVF-ET (preconception screening)	32 (23.5)
Non–IVF-ET (guideline-concordant)	104 (76.5)
Comorbidities, n (%)	
Rheumatoid arthritis	15 (11.0)
Ankylosing spondylitis	15 (11.0)
Concurrent methotrexate use	3 (2.2)
Baseline laboratory values, median (IQR)	
Alanine aminotransferase, U/L	20 (15–31)
Total bilirubin, μmol/L	19.1 (14.8–24.2)
Creatinine, μmol/L	52.6 (45.4–67.1)
White blood cell count, ×10^9^/L	5.17 (4.23–6.26)
Absolute neutrophil count, ×10^9^/L	2.76 (2.24–3.68)
Hemoglobin, g/L	135 (127–147)
Platelet count, ×10^9^/L	248 (210–296)

Abbreviations: IQR, interquartile range; IVF-ET, in vitro fertilization–embryo transfer; TPT, tuberculosis preventive treatment.

### Treatment Completion

**Figure 2.**
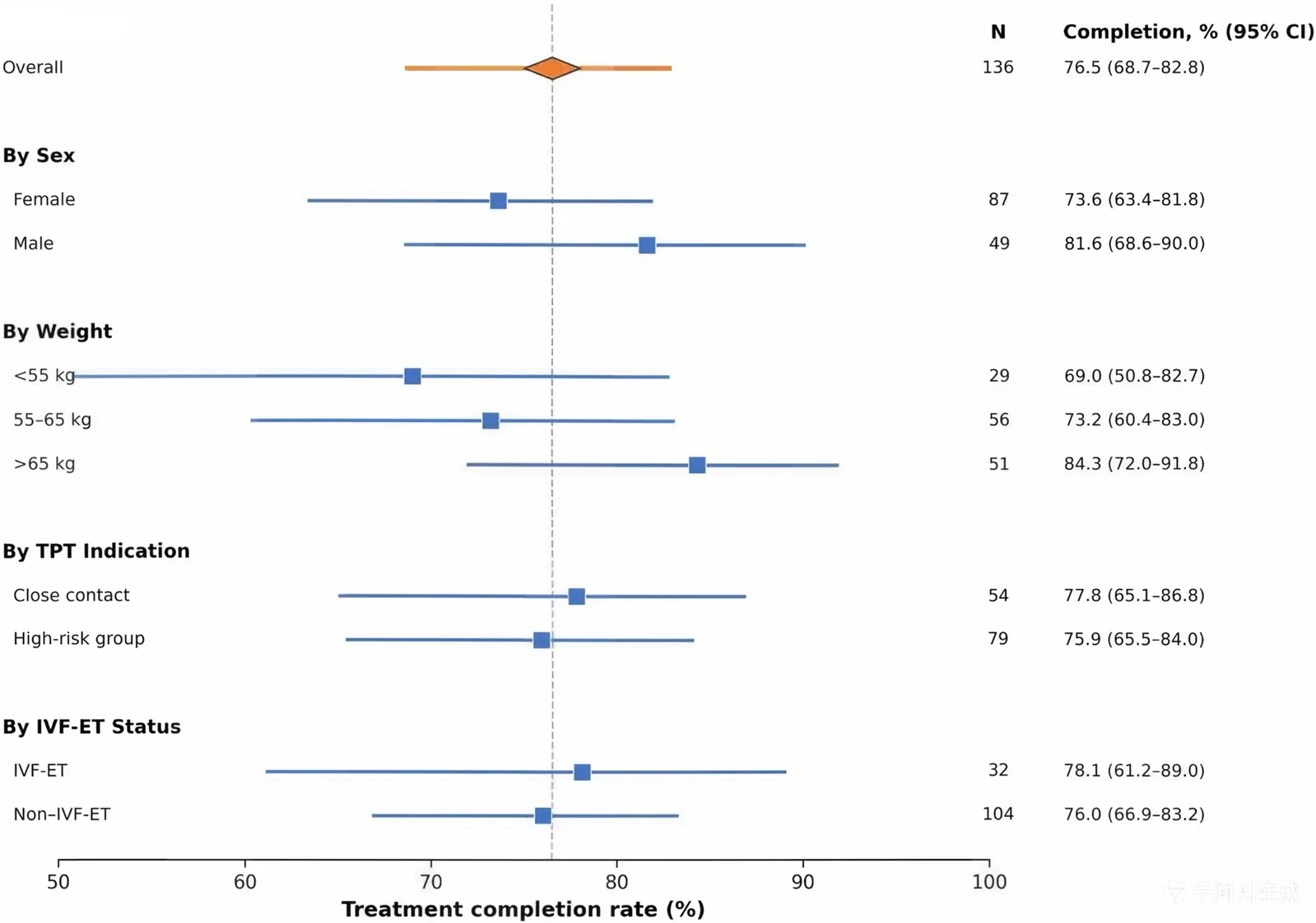
Treatment completion rates of 1HP among participants stratified by baseline demographic and clinical characteristics. Forest plot showing the proportion of participants completing fixed-dose 1HP therapy. Completion rates are presented with 95% confidence intervals for the overall cohort (N = 136) and stratified by sex, body weight, TPT indication, and IVF-ET status. Numbers to the right of the plot indicate the number of participants in each stratum. Abbreviations: CI, confidence interval; IVF-ET, in vitro fertilization–embryo transfer; TPT, tuberculosis preventive treatment.

Treatment completion rates among participants stratified by baseline demographic and clinical characteristics were presented in Figure 2. Of 136 participants, 104 (76.5%; 95% CI, 68.7%–82.8%) completed treatment. Completion rates were consistent across participants with different TPT indications: 77.8% among household contacts and 78.1% among individuals undergoing preconception screening. In sensitivity analyses restricted to guideline-concordant indications, completion remained similar at 76.0% (79/104; 95% CI, 66.9%–83.2%) (Supplementary Table 2).

**Figure 3.**
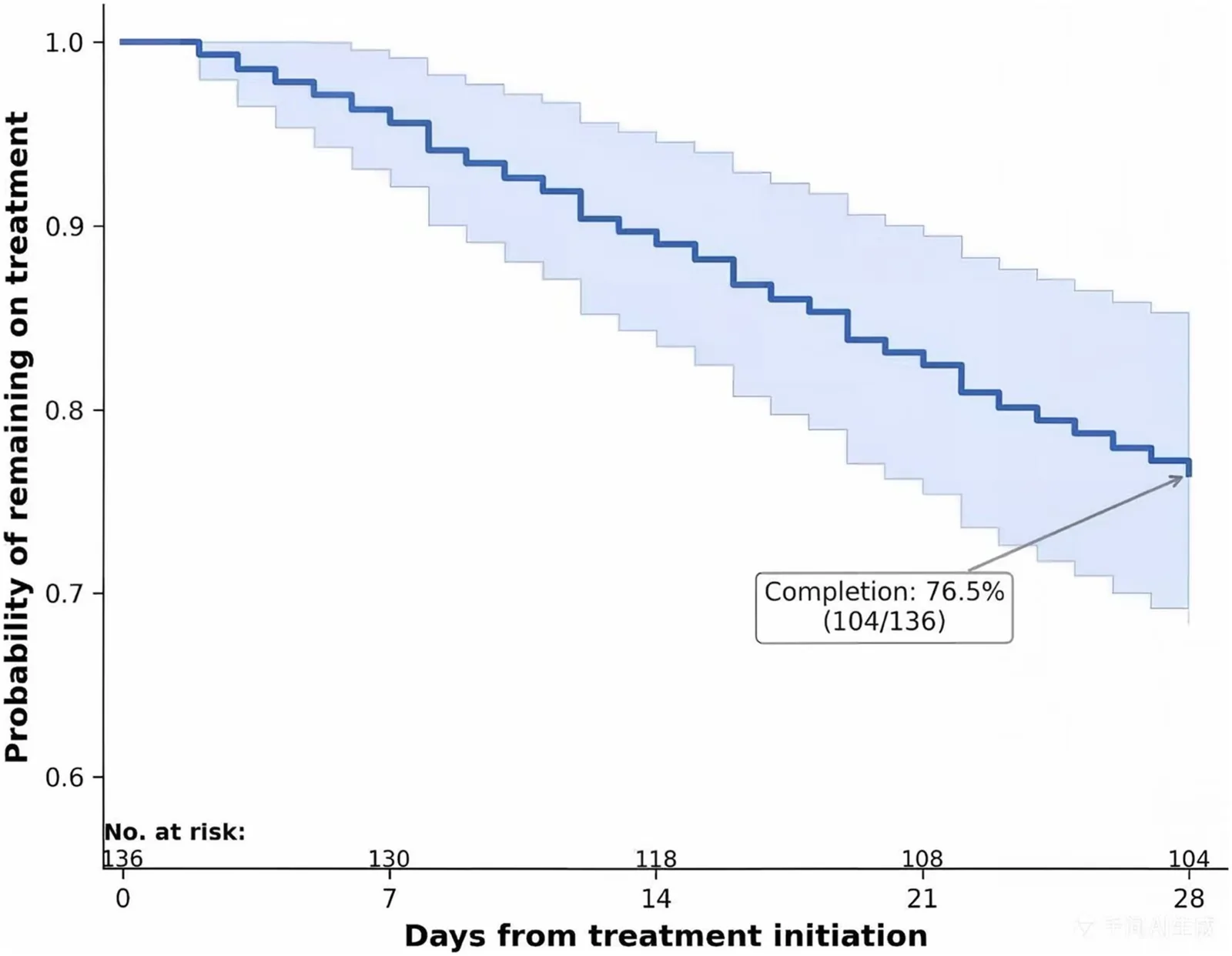
Kaplan–Meier estimate of treatment continuation. Kaplan–Meier curve depicting the probability of remaining on treatment over the 28-day treatment period. The shaded area represents the 95% confidence interval. Vertical tick marks indicate censoring at treatment completion (day 28). The probability of treatment continuation declined progressively from 100% at day 0, with discontinuations distributed throughout the treatment period. The final treatment completion was 76.5% (104/136; 95% CI, 68.7%–82.8%). Numbers at risk are displayed below the x-axis at weekly intervals. Among noncompleters (n = 32), the median time to discontinuation was 12 d (IQR, 8–17 d), indicating that most tolerability-related decisions occurred within the first 2 weeks—a pattern consistent with temporal observations in our pediatric cohort [11]. Abbreviations: CI, confidence interval; IQR, interquartile range.

Among the 32 participants who did not complete treatment, adverse events accounted for most discontinuations (75.0%), followed by loss to follow-up (15.6%) and voluntary withdrawal (9.4%) (Figure 1). The median time to discontinuation was 12 days (IQR, 8–17), indicating that most tolerability-related discontinuations occurred within the first 2 weeks (Figure 3; Supplementary Figure 1).

### Adverse Events

Thirty-six participants (26.5%) experienced at least 1 adverse event (Table 2). Hematologic abnormalities were the most frequent, with neutropenia occurring in 20 participants (14.7%), 6 of whom also developed concurrent leukopenia. Cutaneous reactions were the second most common, with rash affecting 6.6% (9/136), but no one received any specific treatment. Gastrointestinal symptoms were infrequent. The vast majority of adverse events were mild or moderate: 94.4% (34/36) were classified as grades 1–2 according to CTCAE v5.0. Hepatotoxicity occurred rarely (2.9% with alanine aminotransferase exceeding 1.5× baseline), and no participant experienced alanine aminotransferase elevations beyond 3 times the upper limit of normal. No participants with grades 1–2 adverse events received any treatment. Adverse events leading to treatment discontinuation were presented in Supplementary Table 3.

**Table 2.** Adverse Events During 1HP Treatment (N = 136)

Adverse Event	Total, N (%)	Grade 1	Grade 2	Grade ≥ 3
Any adverse event	36 (26.5)	15	19	2
Hematologic				
Neutropenia	20 (14.7)	10	8	2^a^
Leukopenia	6 (4.4)	4	2	0
Dermatologic				
Rash	9 (6.6)	0	9	0
Constitutional				
Fever	2 (1.5)	0	2	0
Fatigue	1 (0.7)	1	0	0
Gastrointestinal				
Nausea	1 (0.7)	1	0	0
Vomiting	1 (0.7)	1	0	0
Neurologic				
Dizziness	2 (1.5)	2	0	0
Psychiatric				
Anxiety	1 (0.7)	0	1	0

Adverse events graded per Common Terminology Criteria for Adverse Events version 5.0 [14].

Abbreviation: ANC, absolute neutrophil count.

^a^Both grade ≥ 3 events (grade 3 neutropenia, ANC < 1.0 × 10^9^/L) occurred in methotrexate users without folic acid supplementation; see Supplementary Discussion for detailed clinical characteristics and proposed mechanisms.

Temporal patterns of adverse events varied by organ system: cutaneous reactions appeared earlier (median day 7; IQR, 5–10), while hematologic toxicity emerged later (median day 14; IQR, 10–17). Grade 3 adverse events occurred in 2 participants (1.5%), both representing grade 3 neutropenia in methotrexate users lacking folic acid supplementation. One participant received single administration of granulocyte colony-stimulating factor (G-CSF), and another declined to received treatment; their neutrophil count normalized within 2 weeks during follow-up evaluation. The single methotrexate recipient who received concurrent folic acid experienced only grade 2 neutropenia and completed treatment successfully. Detailed clinical characteristics and mechanistic considerations appear in Supplementary Tables 4 and Figure 2 and the Supplementary Discussion. All affected participants recovered without infectious complications.

### Rifapentine Pharmacokinetics

Pharmacokinetic analysis included 267 samples collected from 136 participants across 4 scheduled time points (weeks 1–4). The median rifapentine 12-hour postdose concentration was 21.8 mg/L (IQR, 14.8–28.4; range, 5.4–47.5), with an interindividual coefficient of variation of 42.0%. All participants achieved concentrations exceeding 5 mg/L, and 91.9% (125/136) exceeded the model-informed threshold of 10 mg/L (Table 3; Supplementary Table 5).

**Table 3.** Rifapentine Pharmacokinetic Parameters by Body Weight Category

Parameter	Overall (N = 136)	<55 kg (n = 29)	55–65 kg (n = 56)	>65 kg (n = 51)
C_12h_, mg/L, median (IQR)	21.8 (14.8–28.4)	24.1 (14.4–31.2)	23.9 (16.7–28.4)	19.2 (14.5–23.6)
C_12h_ range, mg/L	5.4–47.5	8.6–47.5	6.6–47.1	5.4–37.3
CV, %	42.0	46.3	38.9	37.7
≥10 mg/L^a^, n (%)	125 (91.9)	27 (93.1)	52 (92.9)	46 (90.2)

Abbreviations: C_12h_, 12-h postdose trough concentration; CV, coefficient of variation; IQR, interquartile range.

Per-participant median concentrations (n = 136 participants contributing 267 total samples).

^a^The 10 mg/L threshold represents a model-informed exploratory benchmark derived from population pharmacokinetic modeling [9]; it has not been prospectively validated against clinical outcomes. Pharmacokinetic methodology was validated in our companion pediatric study [11].

**Figure 4.**
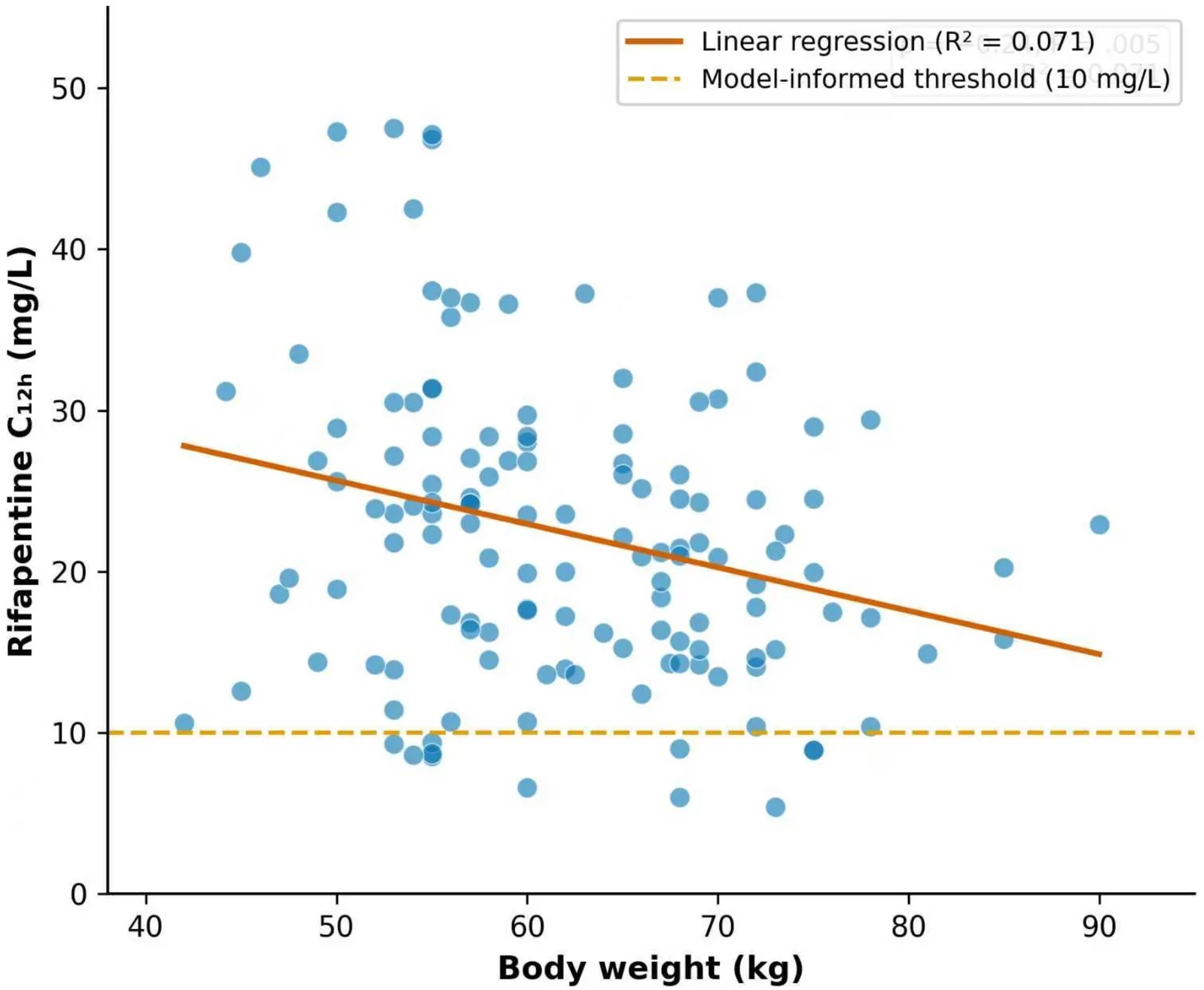
Relationship between body weight and rifapentine concentration. Scatter plot showing the relationship between body weight (x-axis, kg) and per-participant median rifapentine 12-h postdose concentration (y-axis, mg/L) in 136 participants. Each point represents one participant's median concentration calculated from up to 4 samples. The solid line indicates the linear regression fit (slope = −0.27 mg/L per kg); body weight demonstrated a statistically significant but modest inverse correlation with concentration (Spearman ρ = −0.24, *P* = .005), accounting for only 7.1% of interindividual variability (R^2^ = 0.071). The dashed horizontal line indicates the model-informed exploratory threshold of 10 mg/L [9]; 91.9% (125/136) of participants achieved concentrations at or above this threshold. Despite the statistical significance, the modest R^2^ indicates that the majority of pharmacokinetic variability (>90%) is attributable to factors other than body weight, supporting the robustness of fixed-dose delivery across age groups.

Body weight showed a modest inverse correlation with rifapentine concentrations (Spearman ρ = −0.24; *P* = .005; Figure 4). Linear regression indicated that weight explained only 7.1% of interindividual variability (R^2^ = 0.071), corresponding to an estimated decrease of 0.27 mg/L per kilogram. Despite the statistical significance, the clinical impact of weight was limited: Median concentrations remained above 10 mg/L across weight strata (<55 kg, 55–65 kg, and >65 kg), with target attainment rates of 93.1%, 92.9%, and 90.2%, respectively (Table 3).

## DISCUSSION

This prospective study provides real-world data on the safety, feasibility, and pharmacokinetics of fixed-dose 1HP in a Chinese adult population with different TPT indications. Fixed-dose 1HP was well tolerated, and the 76.5% completion rate may offer a pragmatic benchmark for programmatic planning. Pharmacokinetic data confirmed adequate rifapentine exposure in 91.9% of participants, and body weight explained only 7.1% of variability in drug concentrations, supporting simplified weight-independent dosing.

Interpretation of the 76.5% completion rate requires attention to implementation context and cross-study differences in both treatment delivery and outcome definitions. BRIEF-TB used direct observed therapy (DOT) and required completion of all 28 directly observed doses [6], while the Ultra-Curto trial used SAT and defined completion as ≥25 doses (approximately 90% adherence) [16]. Our study used SAT with a completion threshold of ≥23 doses (≥80% adherence) within a 40-day window. Given these differences in both treatment delivery and outcome definitions, direct comparisons of completion rates across studies should be made cautiously. The reason that our completion rate (76.5%) was the lowest among these studies may also be explained by differences in follow-up intensity. Our study required weekly visits due to initial safety concerns, potentially increasing participant burden. Once safety is established, less frequent monitoring might streamline follow-up schedules. Further implementation research is needed to assess whether similar or even higher completion rates can be achieved in routine programmatic settings with less intensive follow-up schedules in China.

The consistency of completion rates across participants with different TPT indications—77.8% for household contacts, 78.1% for women preparing for IVF-ET, and 75.9% for immunosuppressive therapy candidates—carries several implications for implementation. This similarity suggests that completion is shaped primarily by modifiable system-level factors, including adverse-event management, follow-up practices, and patient counseling, rather than intrinsic differences among patient groups.

Pharmacokinetic findings provide additional support for simplified dosing. Body weight explained only 7.1% of variability in rifapentine concentrations (R^2^ = 0.071), indicating that anthropometric measures are poor predictors of drug exposure and that pharmacogenetic factors—particularly AADAC polymorphisms—likely play a dominant role [10]. These results align with our companion pediatric cohort, in which weight-based dosing also produced inadequate exposures, particularly in children whose body weight was less than 15 kg [11]. From a practical standpoint, weight-based adjustments would add operational complexity without meaningfully reducing variability. A uniform 600 mg rifapentine dose eliminates the need for scales, dose calculations, and multiple tablet strengths, thereby facilitating task-shifting and addressing supply-chain barriers that have historically limited TPT uptake [7]. Although pharmacogenetic variability was substantial (CV = 42%), it did not prevent 91.9% of participants from achieving the model-informed exposure threshold.

The overall safety profile (26.5% any adverse event, 1.5% grade 3 or higher) compares favorably with established TPT regimens and positions 1HP among well-tolerated short-course options [7]. The neutropenia rate of 14.7% deserves contextual interpretation: The study protocol mandated intensive weekly monitoring with complete blood counts, which may have increased detection of subclinical laboratory abnormalities compared with programmatic settings where monitoring is less frequent. Encouragingly, 90% of neutropenia events were grades 1–2; no participant developed febrile neutropenia, and all cases resolved without infectious complications. The absence of hepatotoxicity (0% with Alanine aminotransaminase 3 times the upper limit of normal, ALT > 3× ULN) is notable given historical concerns about rifamycin–isoniazid combinations and supports the hepatic safety of 1HP in this population.

An exploratory observation warrants comment: Both grade ≥ 3 adverse events occurred in methotrexate users without folic acid supplementation (2/2), whereas the single methotrexate user receiving folic acid completed treatment with only grade 2 neutropenia. While causal inference is precluded by the extremely small sample size (n = 3), this pattern is biologically plausible. Rifamycin can inhibit organic anion transporters involved in methotrexate elimination [17], and methotrexate's inhibition of dihydrofolate reductase, which depletes folate for DNA synthesis, can be mitigated by folic acid supplementation [18, 19]. Enhanced hematologic monitoring and folic acid supplementation might be of value when 1HP is coadministered with methotrexate until confirmatory data from larger studies are available. Detailed clinical characteristics and mechanistic hypotheses appear in the Supplementary Discussion.

There are several limitations of our study. Firstly, the single-center design may limit generalizability, although the heterogeneous enrollment enhances relevance for implementation settings. Inclusion of participants with different TPT indications strengthens external validity but limits direct comparison with trials restricted to homogeneous guideline-concordant populations. Secondly, the intensive weekly monitoring protocol may have increased adverse-event ascertainment relative to routine programmatic practice, where laboratory testing is less frequent. Consequently, the safety profile in typical settings may therefore appear more favorable. Thirdly, the 10 mg/L threshold is a model-informed benchmark rather than a clinically validated therapeutic target and should be interpreted accordingly. The clinical significance of specific trough concentrations for preventive efficacy remains incompletely characterized, and our use of this threshold is intended to facilitate comparison with prior pharmacokinetic studies rather than to define therapeutic success or failure. Similarly, 12-hour postdose sampling provides a pragmatic but midinterval measure rather than a true 24-hour trough concentration.

Despite these limitations, the core finding is robust: A fixed 600 mg rifapentine dose achieved model-informed target concentrations across the 42–90 kg weight range in this prospectively enrolled East Asian cohort, with acceptable completion and good tolerability. These results address a key evidence gap highlighted by WHO and support simplified programmatic delivery of 1HP.

## CONCLUSIONS

In this prospective implementation study among Chinese adults receiving TPT, fixed-dose 1HP demonstrated good safety and feasibility, with consistent pharmacokinetic exposure across a broad weight range and 91.9% of participants reaching the model-informed concentration benchmark. These findings support simplified, weight-independent dosing and the real-world scale-up of 1HP in tuberculosis-endemic settings, including East Asian populations with lower median body weight.

## Supplementary Material

ciag247_Supplementary_Data

## Supplementary Data

Supplementary materials are available at *Clinical Infectious Diseases* online. Consisting of data provided by the authors to benefit the reader, the posted materials are not copyedited and are the sole responsibility of the authors, so questions or comments should be addressed to the corresponding author.
