# Supplementary material for: Fixed-Dose Rifapentine–Isoniazid (1HP) for Tuberculosis Preventive Treatment in Chinese Adults: A Prospective Real-World Safety and Pharmacokinetic Study
Source: Clin Infect Dis. 2026 Apr 13;83(2):e386–94. doi: 10.1093/cid/ciag247 (PMC13537151; doi:10.1093/cid/ciag247)
Supplement: Supplementary file 1 [file ciag247_supplementary_data.docx]

**SUPPLEMENTARY MATERIALS**

*Fixed-Dose Rifapentine–Isoniazid (1HP) for Tuberculosis Preventive Treatment in Chinese Adults: A Prospective Real-World Safety and Pharmacokinetic Study*

**Contents**

Supplementary Methods S1. LC-MS/MS Assay Validation

Supplementary Methods S2. Statistical Analysis Details

Supplementary Table S1. Inclusion and Exclusion Criteria of 1HP for TBI

Supplementary Table S2. Sensitivity Analysis: Treatment Completion by different TPT indication

Supplementary Table S3. Adverse Events Leading to Treatment Discontinuation

Supplementary Table S4. Characteristics and Outcomes of Methotrexate Users

Supplementary Table S5. Rifapentine Pharmacokinetic Parameters by Sampling Timepoint

Supplementary Figure S1. Treatment Timeline (Swimmer Plot)

Supplementary Figure S2. Absolute Neutrophil Count Trajectories in Methotrexate Users Supplementary Discussion: Hypothesis-Generating Observations on Methotrexate-Rifapentine Interaction

# Supplementary Methods S1. LC-MS/MS Assay Validation

Rifapentine plasma concentrations were measured using a validated liquid chromatography–tandem mass spectrometry (LC-MS/MS) method. The assay was developed and validated according to FDA bioanalytical method validation guidance, with complete methodological details and validation parameters published in our companion pediatric study [11].

**Assay Summary**

Plasma samples (100 μL) were prepared using protein precipitation with acetonitrile containing rifapentine-d3 as internal standard. Chromatographic separation was performed on an Agilent Poroshell 120 EC-C18 column (2.1 × 50 mm, 2.7 μm) maintained at 40°C with gradient elution (mobile phase: 0.1% formic acid in water/acetonitrile). Detection employed a Shimadzu LC-MS 8050 system with electrospray ionization in positive ion mode. Multiple reaction monitoring transitions were: rifapentine m/z 877.4 → 845.4 (quantifier); rifapentine-d3 m/z 880.4 → 848.4 (internal standard).

**Validation Summary**

The validated concentration range was 0.1–100 mg/L. Intra-day precision (CV) ranged from 2.8% to 5.6%; inter-day precision ranged from 3.2% to 6.1%. Accuracy ranged from 96.4% to 103.8%. The lower limit of quantification was 0.1 mg/L with signal-to-noise ratio >10. Stability was confirmed under bench-top (4 hours at room temperature), freeze-thaw (3 cycles), and long-term storage (−80°C for 6 months) conditions.

For complete validation data including calibration curves, quality control results, and specificity assessments, see Liu et al. [11].

# Supplementary Methods S2. Statistical Analysis Details

**Pharmacokinetic Analysis**

Rifapentine trough concentrations (C₁₂ₕ) were defined as plasma concentrations measured 12 ± 2 hours after the preceding dose. For participants with multiple concentration measurements, the per-participant median was calculated to provide a single representative value for correlation analyses, ensuring independence of observations. This approach reduces the influence of outlier measurements while maintaining the full dataset for descriptive statistics. The 10 mg/L model-informed threshold was derived from population pharmacokinetic modeling by Hibma et al. [9], who demonstrated through simulations that fixed-dose rifapentine 600 mg achieves adequate exposure across body weight ranges. This threshold has not been prospectively validated against clinical endpoints and should be interpreted as a pharmacokinetic benchmark rather than a validated therapeutic target.

**Statistical Methods**

Continuous variables were summarized as median with interquartile range (IQR) given the non-normal distribution of pharmacokinetic data confirmed by Shapiro-Wilk testing. Categorical variables were expressed as counts with percentages. Correlation between body weight and rifapentine concentration was assessed using Spearman rank correlation (ρ) to avoid assumptions of linearity; linear regression was additionally performed to estimate the coefficient of determination (R²) for comparison with prior literature. The coefficient of variation (CV) was calculated as (standard deviation / mean) × 100%. Group comparisons for continuous variables used the Mann-Whitney U test (2 groups). Categorical variables were compared using Fisher exact test given small expected cell counts. Two-sided P values <.05 were considered statistically significant. No adjustment for multiple comparisons was performed given the exploratory nature of subgroup analyses; findings should be interpreted as hypothesis-generating.

**Confidence Intervals**

Wilson score intervals were used for proportions to provide appropriate coverage even for proportions near 0 or 1 and for small sample sizes.

**Power Considerations**

With only 3 methotrexate users, statistical power was approximately 25% to detect a threefold increase in grade ≥3 neutropenia risk at α = 0.05; findings in this subgroup are explicitly exploratory and hypothesis-generating.

# Supplementary Table S1. Inclusion and Exclusion Criteria of 1HP for TBI

| Category | Criteria |
| --- | --- |
| Inclusion Criteria | |
| 1 | Age ≥18 years |
| 2 | Confirmed tuberculosis infection (positive interferon-gamma release assay [IGRA]) |
| 3 | No evidence of active tuberculosis (based on symptom screening, chest radiography, and, if indicated, computed tomography) |
| 4 | At least one of the following high-risk criteria for progression to active TB: • Household contact of a patient with bacteriologically confirmed pulmonary tuberculosis  (screening within one month of index case diagnosis) • Planned initiation of immunosuppressive therapy, including tumor necrosis factor-α inhibitors,  other immunosuppressants, or systemic corticosteroids (equivalent to prednisone ≥15 mg/day for ≥1 month) • Preparation for in vitro fertilization–embryo transfer (IVF-ET) in a high-incidence setting |
| 5 | Willing and able to provide written informed consent |
| Exclusion Criteria | |
| 1 | Known hypersensitivity to rifapentine, isoniazid, or any related drugs |
| 2 | Active hepatitis or baseline alanine aminotransferase (ALT) >3 times the upper limit of normal |
| 3 | Estimated glomerular filtration rate <30 mL/min/1.73 m² |
| 4 | Concurrent use of medications with significant drug–drug interactions  (e.g., protease inhibitors, certain anticonvulsants) |
| 5 | Pregnancy or breastfeeding |
| 6 | Any condition that, in the investigator’s judgment, would compromise participant safety or study adherence |

# Supplementary Table S2. Sensitivity Analysis: Treatment Completion by different TPT indication

| **Subgroup** | **n** | **Completed, n (%)** | **95% CI** |
| --- | --- | --- | --- |
| Overall population | 136 | 104 (76.5) | 68.7–82.8 |
| **By TPT indication** |  |  |  |
| Close contact only | 54 | 42 (77.8) | 65.1–86.8 |
| High-risk group only | 79 | 60 (75.9) | 65.5–84.0 |
| Both indications | 3 | 2 (66.7) | 20.8–93.9 |
| **By IVF-ET status** |  |  |  |
| IVF-ET (pre-conception) | 32 | 25 (78.1) | 61.2–89.0 |
| Non–IVF-ET (guideline-concordant) | 104 | 79 (76.0) | 66.9–83.2 |
| **By weight category** |  |  |  |
| <55 kg | 29 | 20 (69.0) | 50.8–82.7 |
| 55–65 kg | 56 | 41 (73.2) | 60.4–83.0 |
| >65 kg | 51 | 43 (84.3) | 72.0–91.8 |
| **By sex** |  |  |  |
| Female | 87 | 64 (73.6) | 63.4–81.7 |
| Male | 49 | 40 (81.6) | 68.6–90.0 |

*Abbreviations: CI, confidence interval; IVF-ET, in vitro fertilization–embryo transfer; TPT, tuberculosis preventive treatment.* 95% confidence intervals calculated using Wilson score method.

# Supplementary Table S3. Adverse Events Leading to Treatment Discontinuation (n = 24)

| **Adverse Event Type** | **n (%)** | **Median Day** | **Range** | **Grade ≥3** |
| --- | --- | --- | --- | --- |
| Neutropenia | 10 (41.7) | 14 | 7–21 | 2 |
| Rash/dermatologic reaction | 7 (29.2) | 7 | 3–14 | 0 |
| Hepatotoxicity (ALT elevation) | 3 (12.5) | 21 | 14–28 | 0 |
| Gastrointestinal intolerance | 2 (8.3) | 10 | 7–14 | 0 |
| Leukopenia (concurrent with neutropenia) | 2 (8.3) | 14 | 14–21 | 0 |
| **Total** | **24 (100)** | **—** | **—** | **2** |

*Abbreviation: ALT, alanine aminotransferase.* Adverse events graded per CTCAE v5.0. Both grade ≥3 events were grade 3 neutropenia in methotrexate users without folic acid supplementation.

# Supplementary Table S4. Characteristics and Outcomes of Methotrexate Users (n = 3)

| **Characteristic** | **Patient A** | **Patient B** | **Patient C** |
| --- | --- | --- | --- |
| Underlying condition | Lymphoma | Rheumatoid arthritis | Rheumatoid arthritis |
| Methotrexate dose | 10 mg qd | 5 mg qw | 10 mg qw |
| Folic acid supplementation | None | None | Yes (0.4 mg/day) |
| Baseline ANC (×10⁹/L) | 2.50 | 3.20 | 3.80 |
| Nadir ANC (×10⁹/L) | 0.95 | 0.85 | 1.31 |
| Day of nadir | Day 7 | Day 21 | Day 27 |
| Neutropenia grade | Grade 3 | Grade 3 | Grade 2 |
| Rifapentine C₁₂ₕ (mg/L) | 46.8 | 36.9 | 23.6 |
| Treatment completion | No (Day 7) | No (Day 21) | Yes |
| ANC recovery time | 10 days | 14 days | N/A |
| Infectious complications | None | None | None |

*Abbreviations: ANC, absolute neutrophil count; C₁₂ₕ, 12-hour post-dose trough concentration; N/A, not applicable; qd, once daily; qw, once weekly.* Neutropenia graded per CTCAE v5.0: Grade 2, ANC 1.0–1.5 × 10⁹/L; Grade 3, ANC <1.0 × 10⁹/L. Patient C, who received concurrent folic acid supplementation, completed treatment with only grade 2 neutropenia.

# Supplementary Table S5. Rifapentine Pharmacokinetic Parameters by Sampling Timepoint

| **Parameter** | **Week 1** | **Week 2** | **Week 3** | **Week 4** |
| --- | --- | --- | --- | --- |
| Samples, n | 91 | 70 | 45 | 61 |
| C₁₂ₕ, mg/L, median (IQR) | 23.1 (15.1–32.1) | 21.8 (13.8–28.4) | 19.8 (14.6–25.8) | 22.0 (14.1–25.7) |
| C₁₂ₕ range, mg/L | 5.3–54.8 | 5.2–49.2 | 6.6–45.1 | 6.3–37.7 |
| ≥10 mg/L, n (%) | 83 (91.2) | 65 (92.9) | 41 (91.1) | 54 (88.5) |
| CV, % | 44.9 | 45.4 | 42.1 | 38.3 |

C₁₂ₕ denotes 12-hour post-dose rifapentine concentration. CV, coefficient of variation; IQR, interquartile range. Total samples: 267 from 136 participants.

# Supplementary Figure S1. Treatment Timeline (Swimmer Plot)

Swimmer plot displaying individual treatment timelines stratified by treatment outcome.

**Panel A (Non-completers, n = 32):** Horizontal bars represent participants who discontinued treatment before day 28, colored by reason for discontinuation: orange/red indicates adverse events (n = 24), yellow indicates lost to follow-up (n = 5), gray indicates voluntary withdrawal (n = 3). Bar length corresponds to treatment duration until discontinuation. Participants are sorted by treatment duration.

**Panel B (Completers with adverse events, n = 12):** Blue bars indicate participants who completed the full 28-day treatment course despite experiencing adverse events. All bars extend to day 28.

Black star symbols (★) denote participants receiving concurrent methotrexate therapy; two of three methotrexate users appear in Panel A (non-completers at days 7 and 21) and one in Panel B (completer with folic acid supplementation). The vertical dashed line at day 28 marks the intended treatment completion. Note: Sixty-eight participants who completed treatment without adverse events are not displayed.

# Supplementary Figure S2. Absolute Neutrophil Count Trajectories in Methotrexate Users

Temporal patterns of absolute neutrophil count (ANC) during 1HP treatment in the three participants receiving concurrent methotrexate.

**Patient A** (red circles, lymphoma, methotrexate 10 mg daily, no folic acid) developed grade 3 neutropenia by day 7 (nadir ANC 0.95 × 10⁹/L) and discontinued treatment; rifapentine concentration was markedly elevated (46.8 mg/L, 2.2× population median of 21.8 mg/L).

**Patient B** (orange squares, rheumatoid arthritis, methotrexate 5 mg weekly, no folic acid) developed grade 3 neutropenia by day 21 (nadir ANC 0.85 × 10⁹/L) and discontinued treatment; rifapentine concentration was elevated (36.9 mg/L, 1.7× population median).

**Patient C** (blue triangles, rheumatoid arthritis, methotrexate 10 mg weekly, folic acid 0.4 mg daily) completed treatment with grade 2 neutropenia only (nadir ANC 1.31 × 10⁹/L); rifapentine concentration was near the population median (23.6 mg/L).

Dashed horizontal lines indicate CTCAE v5.0 thresholds [14] for grade 2 (1.5 × 10⁹/L, orange) and grade 3 (1.0 × 10⁹/L, red) neutropenia. Light green shading indicates normal ANC range (2.0–7.5 × 10⁹/L). X symbols mark treatment discontinuation.

*Abbreviations: ANC, absolute neutrophil count; CTCAE, Common Terminology Criteria for Adverse Events.*

# Supplementary Discussion: Hypothesis-Generating Observations on Methotrexate-Rifapentine Interaction

**Clinical Observation Summary**

Among three participants receiving concurrent methotrexate during 1HP treatment, a notable pattern emerged: both patients without folic acid supplementation developed grade 3 neutropenia (ANC <1.0 × 10⁹/L) and discontinued treatment, while the single patient receiving daily folic acid (0.4 mg) completed treatment with grade 2 neutropenia only. Although the sample size precludes statistical inference (n = 3, statistical power ~25%), this observation warrants discussion given its potential clinical implications.

**Proposed Mechanistic Framework**

Several non-mutually exclusive mechanisms may underlie this observation:

*Mechanism 1: Organic Anion Transporter (OAT) Inhibition*

Methotrexate undergoes renal elimination primarily via organic anion transporters OAT1 (SLC22A6) and OAT3 (SLC22A8) in the proximal tubule [22]. Rifamycins and their metabolites inhibit these transporters, potentially impairing methotrexate clearance and elevating systemic exposure. While direct evidence for rifapentine-OAT interactions is limited, its structural similarity to rifampicin—a known OAT inhibitor—supports the plausibility of this mechanism. Supporting this hypothesis, the two affected patients exhibited markedly elevated rifapentine trough concentrations (46.8 and 36.9 mg/L) compared with the population median (21.8 mg/L), representing 2.2-fold and 1.7-fold elevations respectively.

*Mechanism 2: Folate Depletion Convergence*

Methotrexate inhibits dihydrofolate reductase (DHFR), depleting cellular folate pools required for DNA synthesis in rapidly proliferating bone marrow cells [23]. The protective effect of folic acid supplementation in Patient C aligns with established rescue therapy principles: exogenous folate can bypass DHFR-dependent pathways, providing tetrahydrofolate directly to cells and mitigating methotrexate-induced myelotoxicity [24].

**Clinical Implications**

These observations, while preliminary, suggest several considerations for patients requiring concurrent methotrexate and 1HP: (1) Folic acid supplementation: Verify that all methotrexate users receive concurrent folic acid (≥0.4 mg daily) before initiating 1HP. (2) Enhanced hematologic monitoring: Consider more frequent complete blood count monitoring (e.g., twice weekly rather than weekly) during the first three weeks. (3) Dose modification consideration: For patients on high-dose methotrexate without folic acid rescue, temporary methotrexate interruption during 1HP may warrant consideration.

**Limitations and Research Recommendations**

The extremely small sample size (n = 3) provides only hypothesis-generating observations. Methotrexate concentrations were not measured, and the proposed pharmacokinetic interaction remains unconfirmed. Prospective studies with adequate statistical power are needed before definitive clinical recommendations can be made.

# Supplementary References

Note: References are numbered continuously with main text. Main text references [1]–[24]; supplementary-specific citations use same numbering.

Key references cited in Supplementary Materials: • [9] Hibma et al. (PK modeling) • [11] Liu et al. (companion pediatric study) • [16] CTCAE v5.0 • [18] VanWert & Sweet (OAT) • [19] Cronstein & Aune (MTX mechanisms) • [20] Shea et al. (Folic acid Cochrane)

*[End of Supplementary Materials]*
